# Multimodal radiomics of cerebellar subregions for machine learning-driven Alzheimer’s disease diagnosis

**DOI:** 10.3389/fnagi.2025.1679788

**Published:** 2025-10-27

**Authors:** Xinqing Hao, Ying Li, Xiulin Wang, Changjun Ma, Ruichen Liu, Yang Jiao, Chunbo Dong, Jing Liu

**Affiliations:** ^1^Stem Cell Clinical Research Center, The First Affiliated Hospital of Dalian Medical University, Dalian, China; ^2^Dalian Innovation Institute of Stem Cell and Precision Medicine, Dalian, China; ^3^Department of Neurology, The First Affiliated Hospital of Dalian Medical University, Dalian, China

**Keywords:** Alzheimer’s disease, cerebellum, radiomics, machine learning, 3DT1W MRI, [^18^F]FDG PET

## Abstract

**Objective:**

This study aimed to develop a machine learning model based on multimodal radiomics features from cerebellar subregions, utilizing the complementarity of cerebellar structural and metabolic imaging data for accurate diagnosis of Alzheimer’s disease (AD).

**Methods:**

A total of 164 cognitively normal (CN) subjects and 146 AD patients from the Alzheimer’s Disease Neuroimaging Initiative (ADNI) database were included. All participants had 3DT1-weighted magnetic resonance imaging (3DT1W MRI) and [^18^F]fluorodeoxyglucose positron emission tomography ([^18^F]FDG PET) imaging data. The cerebellum was divided into 26 subregions, and radiomics features were extracted from different cerebellar regions of these two modality images, respectively. After feature selection, single-modality ([^18^F]FDG PET, 3DT1W MRI) and multimodal ([^18^F]FDG PET + 3DT1W MRI) random forest classification models were constructed. Model performance and clinical value were assessed using area under the curve (AUC), calibration curves, and decision curve analysis (DCA). In addition, we also used Shapley Additive exPlanations (SHAP) to clarify the contributions of features, thereby enhancing the interpretability of the model.

**Results:**

All three models could effectively diagnose AD, with the multimodal model showing the best performance. In the independent test set, the multimodal model achieved an AUC of 0.903, which was higher than the single-modality models based on [^18^F]FDG PET (AUC = 0.842) and 3DT1W MRI (AUC = 0.804). The calibration curves and DCA demonstrated that all three models had good calibration and clinical applicability, especially the multimodal model. SHAP analysis of the multimodal model revealed that among the 15 selected features, the top seven features with the highest SHAP values were derived from [^18^F]FDG PET images, with R_FDG_CER_III_original_firstorder_90Percentile and R_FDG_CER_VI_original_firstorder_Median being the two most important features for distinguishing AD from CN.

**Conclusion:**

The multimodal radiomics model based on cerebellar subregions, which integrates [^18^F]FDG PET and 3DT1W MRI data, can effectively diagnose AD and provide potential biomarkers for clinical applications.

## 1 Introduction

Alzheimer’s disease (AD) is a neurodegenerative disease characterized by progressive cognitive decline. With the aging of the population, the incidence of AD continues to rise, posing a significant threat to global public health (Knopman et al., 2021). The definitive diagnosis of AD relies on invasive autopsy or pathological biopsy. Currently, there is no effective cure for AD, but early intervention can delay disease progression (Crous-Bou et al., 2017). Therefore, the development of non-invasive, highly sensitive biomarkers for the early identification of AD and intervention has become a major focus of current research.

In recent years, the role of the cerebellum in cognitive regulation and emotional responses has received increasing attention, and it may be involved in AD pathology through multiple mechanisms (Lin and Kuo, 2024; Iskusnykh et al., 2024). Structural magnetic resonance imaging (MRI) studies have shown specific cerebellar gray matter atrophy in AD patients, which correlates negatively with cognitive abilities (Toniolo et al., 2018). Functional MRI has further revealed significant disruption in cerebellar-cortical functional connectivity in AD patients, particularly within the default mode network and fronto-parietal networks (Tang et al., 2021). Basic research has provided direct evidence for the cerebellum’s critical role in early AD events; for example, abnormal cerebellar electroencephalogram power spectra in APPswe/PS1ΔE9 transgenic mice precede cerebral amyloid-beta (Aβ) deposition and cognitive deficits (Yu et al., 2023). Furthermore, approximately 10 years before the clinical onset of autosomal dominant AD patients, specific deposition of cerebellar Aβ plaques has already occurred in PSEN1 E280A mutation carriers with unimpaired cognition (Ghisays et al., 2021). These findings underscore the potential importance of the cerebellum in AD pathology and suggest it may provide a novel perspective for early diagnosis.

Radiomics involves the high-throughput extraction of quantitative features from medical images, which can reveal pathological changes hidden in traditional imaging and uncover a large amount of deep biological information (Pirozzi et al., 2025). In recent years, radiomics combined with artificial intelligence algorithms has been successfully applied to the diagnosis, differentiation, and prognosis prediction of AD (Kale et al., 2024; Bevilacqua et al., 2023). However, existing studies primarily focus on the whole brain or hippocampus, overlooking the cerebellum, and have the following limitations: (1) the high dimensionality and heterogeneity of whole brain features; (2) most studies are based on single-modality MRI or positron emission tomography (PET), making it difficult to capture multi-dimensional pathological information (Shi et al., 2024). Recent radiomics based on cerebellar 3DT1-weighted MRI (3DT1W MRI) has shown advantages in AD diagnosis (Chen et al., 2025), but it primarily reflects the macrostructural remodeling of brain tissue and may lag behind early pathological events at the molecular level. In contrast, [^18^F] fluorodeoxyglucose positron emission tomography ([^18^F]FDG PET) can directly reflect the functional status of neuronal activity by assessing glucose metabolism in brain regions. Studies have shown that the cerebellar FDG metabolic pattern exhibits dynamic complexity during the AD pathological process: cerebellar metabolism is significantly reduced in severe AD patients (Ishii et al., 1997), while it is compensatorily enhanced in mild to moderate AD patients, contributing to the formation of the characteristic AD metabolic pattern, and shows a high degree of accuracy in distinguishing cognitively normal individuals from other types of dementia (Perovnik et al., 2022a; Perovnik et al., 2022b). Longitudinal studies have further confirmed that cerebellar FDG metabolism is an effective indicator for predicting the conversion of mild cognitive impairment (MCI) to AD (Blazhenets et al., 2019). However, studies on cerebellar FDG metabolism are still limited, and metabolic heterogeneity in different subregions of the cerebellum and its role in AD diagnosis have not been fully explored. It is also unclear whether combining radiomics features of cerebellar metabolism and structure improves the accuracy of early diagnosis.

This study aims to develop a multimodal radiomics model based on the cerebellum, integrating [^18^F]FDG PET and 3DT1W MRI images radiomic features to explore the potential of the cerebellum in AD diagnosis. By segmenting the cerebellum into different subregions and using model visualization techniques, we aim to evaluate the importance of these subregional structural and metabolic radiomics features in diagnostic accuracy. Through this multimodal approach, we hope to provide more comprehensive and sensitive biomarkers for the early diagnosis of AD and provide new insights into the cerebellar pathophysiology of AD.

## 2 Materials and methods

**Figure 1:**
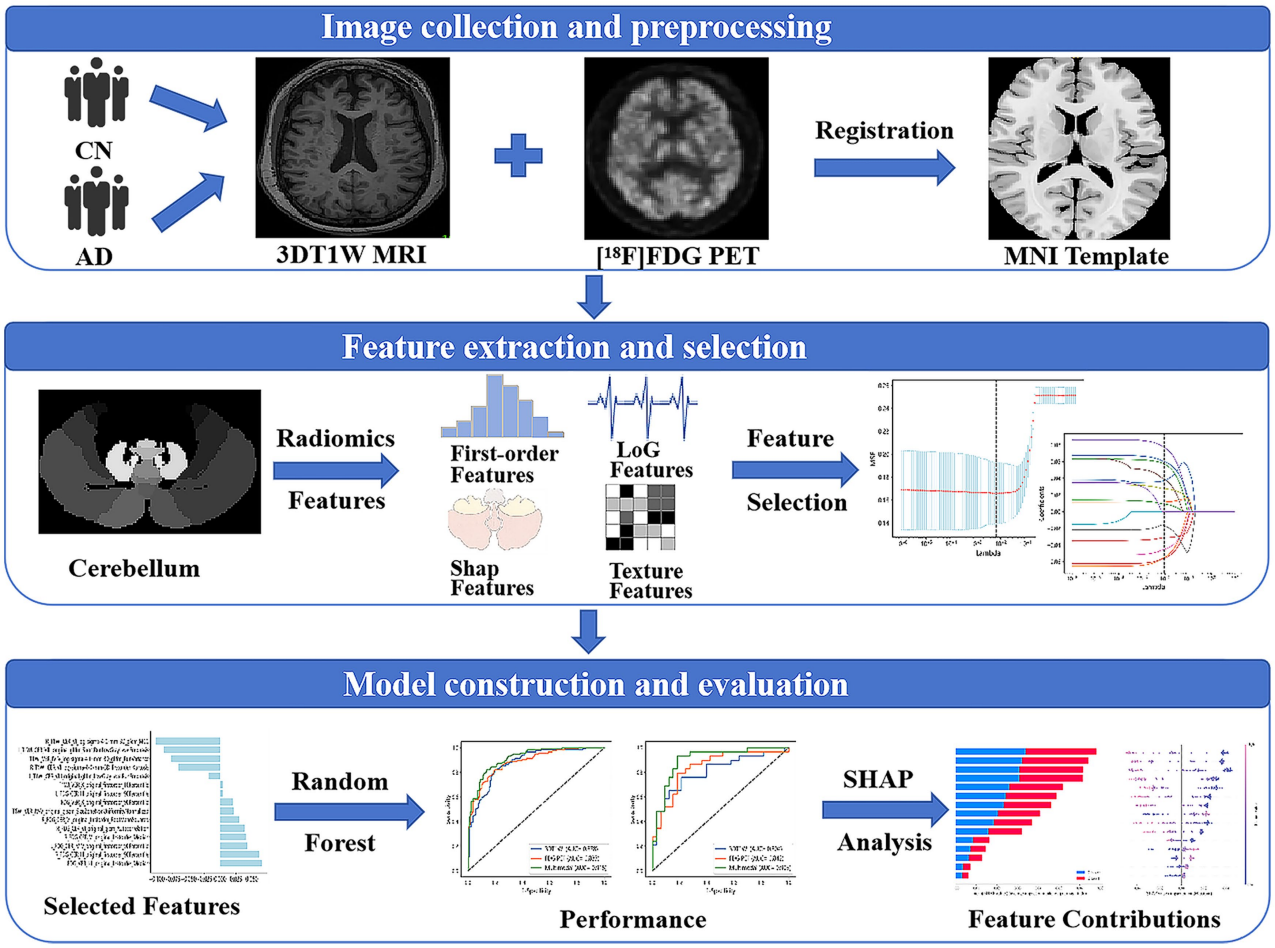
Radiomics workflow. 3DT1W MRI, 3DT1-weighted magnetic resonance imaging; [^18^F]FDG PET, [^18^F]fluorodeoxyglucose positron emission tomography; MNI, Montreal Neurological Institute.

Figure 1 shows the general framework of this study, which primarily includes the following steps: (1) image collection and preprocessing; (2) feature extraction and selection; (3) classification model construction and evaluation, as detailed in the following steps.

### 2.1 Participants

The data used in this study were obtained from the Alzheimer’s Disease Neuroimaging Initiative (ADNI)^1^. ADNI is a large-scale, multicenter study aimed at monitoring the progression of MCI and AD through a series of neuroimaging techniques, neuropsychological assessments, and biomarker analyses. ADNI has received approval from the ethics committees of each participating site, and all participants provided written informed consent.

In this study, 310 ADNI participants were included, consisting of 164 CN and 146 AD subjects from the ADNI1, ADNI2/GO and ADNI3 cohorts. For data collection, all participants were required to have both [^18^F]FDG PET and 3DT1W MRI imaging data, with a scan interval not exceeding 2 weeks to avoid time-related pathological and physiological changes, ensuring the consistency and comparability of the data. Additionally, demographic and genetic information, including sex, age, education level, and APOE genotype, were also collected for these participants.

### 2.2 Image acquisition

The ADNI project online information provides detailed descriptions of [^18^F]FDG PET and 3DT1W MRI acquisition procedures. For the PET images, subjects underwent dynamic 3D scanning in six frames of 5 min each, starting 30–60 min after intravenous injection of 185 MBq (5 mCi) of [^18^F]FDG, with a 30-min interval between frames. For MRI images, T1-weighted structural imaging was acquired using 3DT1 MPRAGE or equivalent protocols with slightly different resolutions. Parameters are slightly different between scanners. The Siemens scanner parameters were: repetition time (TR) = 2,300 ms, matrix = 240 × 256 × 176, slice thickness = 1.2 mm; the General Electric scanner parameters were: TR = 7 ms, matrix = 256 × 256 × 166, slice thickness = 1.2 mm; and the Philips scanner parameters were: TR = 6.8 ms, matrix = 256 × 256 × 170, slice thickness = 1.2 mm.

### 2.3 Imaging preprocessing

To ensure optimal differentiation of imaging features across different subjects, standardized image preprocessing was performed using Statistical Parametric Mapping (SPM12) software (Wellcome Department of Imaging Neuroscience, Institute of Neurology, London, United Kingdom), implemented in MATLAB R2018a (MathWorks Inc., Sherborn, MA, United States). First, MRI and PET images were converted from DICOM format to Neuroimaging Informatics Technology Initiative (NIFTI, nii) format using dcm2niix in MRIcron^2^ for SPM12 compatibility.

For MRI images, Computational Anatomy Toolbox (CAT12) was used to perform skull stripping, N4 bias field correction, normalization to the Montreal Neurological Institute (MNI) space, and smoothing for noise reduction, followed by automatic segmentation into gray matter, white matter, and cerebrospinal fluid. PET images were coregistered with the corresponding MRI images, normalized to the MNI space, and smoothed using an 8 mm isotropic Gaussian kernel. Finally, the normalized PET and MRI images were prepared for input, with a voxel size of 1.0 mm^3^ and dimensions of 161 × 197 × 161.

### 2.4 Cerebellar segmentation and feature extraction

To obtain more detailed cerebellar features, 26 cerebellar regions from the MNI-provided Anatomical Automatic Labeling atlas were used as regions of interest (ROI). These include bilateral cerebellar lobules Crus I, Crus II, III, IV-V, VI, VIIB, VIII, IX, X, as well as vermal lobules I-II, III, IV-V, VI, VII, VIII, IX, X. A total of 200 radiomic features were extracted from each ROI in both [^18^F]FDG PET and 3DT1W MRI images, including 14 shape features, 18 first-order features, 24 gray-level co-occurrence matrix (GLCM) features, 14 gray-level dependence matrix (GLDM) features, 16 gray-level size zone matrix (GLSZM) features, 16 gray-level run length matrix (GLRLM) features, 5 neighboring gray-tone difference matrix (NGTDM) features, and 93 Laplacian of Gaussian (LoG) features. Therefore, a total of 5,200 (200 × 26 = 5,200) features were extracted from each modality.

### 2.5 Feature selection

Systematic preprocessing was performed before feature selection, including addressing outliers and missing values, and eliminating the magnitude differences of multimodal radiomics features by Z-score normalization. To ensure the generalization performance of the classification model, the complete dataset was randomly split into training and test subsets at an 8:2 ratio, with 80% for training and 20% for independent validation. Feature selection was performed on the training set using Python 3.9 software. First, statistically significant differences between groups were characterized by the Mann–Whitney U test (*p* < 0.05). Subsequently, the Maximum Relevance Minimum Redundancy (mRMR) algorithm was employed to remove redundant or irrelevant features, enhancing feature independence. Next, recursive feature elimination (RFE) was used to iteratively eliminate the least contributive features through stepwise backward elimination. Finally, least absolute shrinkage and selection operator (LASSO) regression with 10-fold cross-validation was applied to optimize the regularization parameter *λ*, retaining features with non-zero coefficients to construct the final classification model. Radiomics scores (Rad-Score) were calculated for each subject.

### 2.6 Model construction and evaluation

Using the features selected from the training set, the classification model to discriminate AD from CN was developed using the Random Forest (RF) algorithm, and the generalization performance of the models was evaluated by the independent test set. To investigate the synergistic diagnostic value of multimodal imaging, three distinct classification models were constructed: [^18^F]FDG PET model, 3DT1W MRI model, and multimodal model combining [^18^F]FDG PET and 3DT1W MRI. Model performances were assessed by the following metrics, including the receiver operating characteristic (ROC) curve with calculated area under the curve (AUC), accuracy, sensitivity, specificity, positive predictive value, negative predictive value, and F1 score. The stability of the AUC values was further evaluated using bootstrap resampling to calculate 95% confidence intervals. To further validate reliability, calibration curves were generated to evaluate the consistency of the predicted probabilities with the true labels, while decision curve analysis (DCA) quantified clinical net benefits across risk thresholds. Finally, the Shapley Additive exPlanations (SHAP) interpretability framework was introduced to parse the key feature contributions, thereby revealing the impact of imaging markers on classification decisions.

In addition, to rigorously evaluate whether the model performance was driven by the radiomics features themselves rather than the specific architecture of the Random Forest classifier, we conducted comprehensive robustness validation. Using the same radiomics features and training/test set split, we performed a comparative analysis using seven additional machine learning algorithms, including Logistic Regression (LR), Support Vector Machine (SVM), k-Nearest Neighbors (KNN), Decision Tree (DT), Light Gradient Boosting Machine (LightGBM), eXtreme Gradient Boosting (XGBoost), and Gaussian Naive Bayes (GNB).

### 2.7 Statistical analysis

Demographic differences were analyzed using SPSS 25.0 (IBM SPSS, Chicago, IL, USA). The Shapiro–Wilk test was conducted to assess the normality of the data. Normally distributed continuous variables were compared using independent-sample t-tests, while non-normally distributed continuous variables were analyzed via Mann–Whitney U tests. Categorical data were compared using the chi-square test. *p* < 0.05 was considered statistically significant.

Radiomics machine learning model construction and evaluation were carried out using Python (version 3.9)^3^. Radiomics features were extracted with the pyradiomics package, and machine learning models were developed using scikit-learn. The matplotlib and scikit-learn libraries were used to plot ROC curves, calibration curves, and DCA. Feature importance was calculated and visualized by the shap package, producing SHAP value heatmaps and summary plots.

## 3 Results

### 3.1 Demographic characteristics

A total of 310 participants were included in this study. There were no statistically significant differences in gender (*p* = 0.545) and age (*p* = 0.485) between the two groups. However, significant differences were found between the groups in education (*p* = 0.008), APOE ε4 allele carrier status (*p* < 0.001), and Mini-Mental State Examination (MMSE) scores (*p* < 0.001). Specifically, the AD group exhibited shorter education duration, higher prevalence of APOE ε4 carriers, and lower MMSE scores compared to the CN group. Detailed information is shown in Table 1.

**Table 1:** Demographic data of the CN and AD groups.

	CN (*n* = 164)	AD (*n* = 146)	*p*
Age (years)	77.09 ± 6.52	77.16 ± 7.23	0.485
Gender (M/F)	91/73	76/70	0.545
Education (years)	16.35 ± 2.83	15.45 ± 2.92	0.008^*^
APOE ε4 (+/−)	51/113	106/40	<0.001^*^
MMSE score	29.02 ± 1.17	22.34 ± 4.05	<0.001^*^

CN, cognitively normal; AD, Alzheimer’s disease. *p < 0.05.

The AD and CN groups were randomly divided 8 to 2 into the training and test sets, and Table 2 provides demographic differences within the training and test set groups.

**Table 2:** Demographic data of the training and test set.

	Training set (*n* = 248)			Test set (*n* = 62)		
	CN (*n* = 131)	AD (*n* = 117)	*p*	CN (*n* = 33)	AD (*n* = 29)	*p*
Age (years)	76.95 ± 6.56	77.57 ± 7.29	0.192	77.61 ± 6.45	75.52 ± 6.86	0.243
Gender (M/F)	72/59	60/57	0.562	19/14	16/13	0.849
Education (years)	16.34 ± 2.81	15.49 ± 2.93	0.026^*^	16.36 ± 2.97	14.97 ± 2.81	0.045^*^
APOE ε4 (+/−)	46/85	84/33	<0.001^*^	5/28	22/7	<0.001^*^
MMSE score	28.97 ± 1.20	22.22 ± 4.14	<0.001^*^	29.24 ± 1.06	22.79 ± 3.69	<0.001^*^

CN, cognitively normal; AD, Alzheimer’s disease; MMSE, Mini-Mental State Examination. *p < 0.05.

### 3.2 Feature selection result

**Figure 2:**
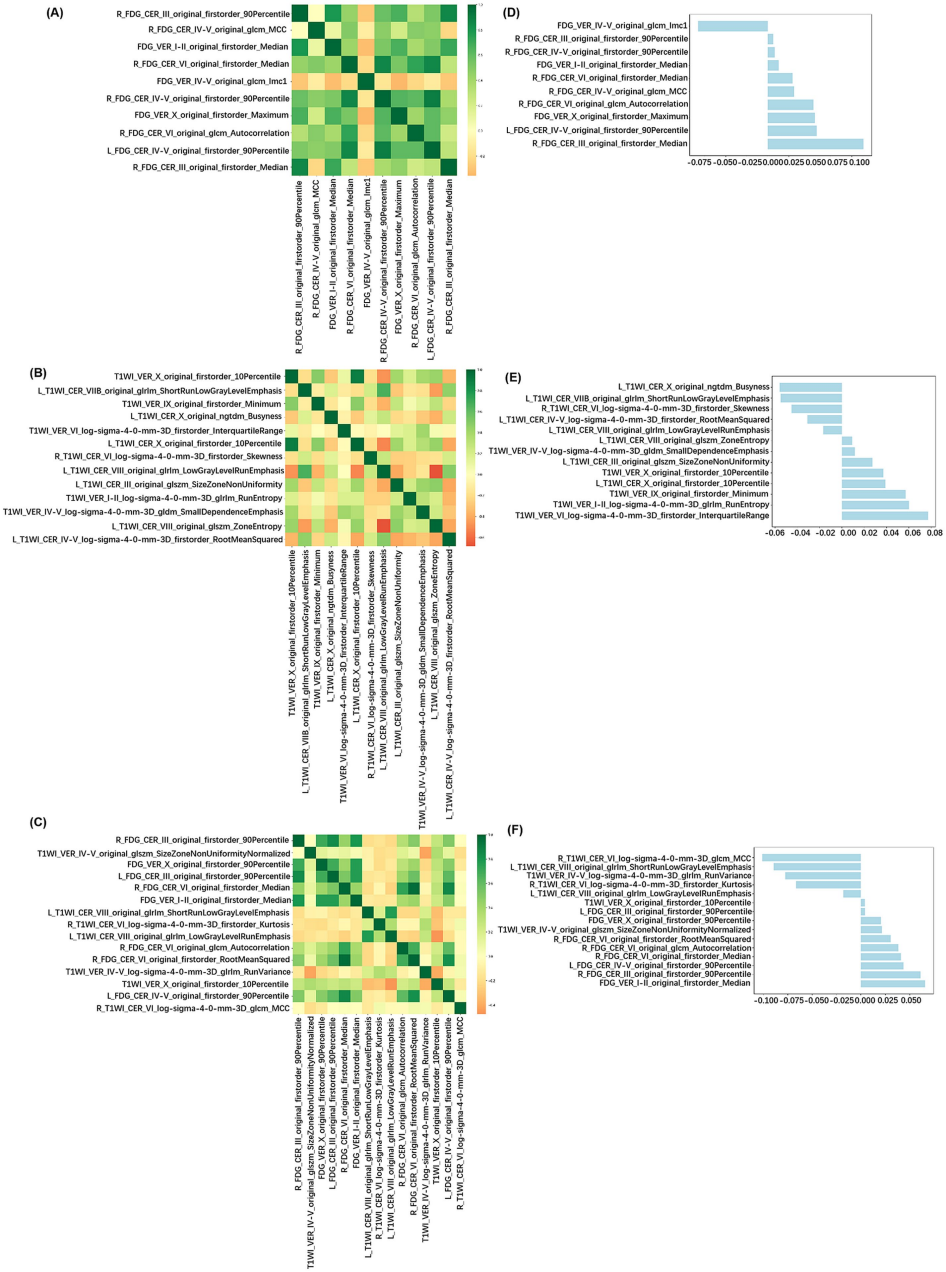
Relationship graphs of radiomics features used for modeling. Correlation heatmap of radiomics features in the [^18^F]FDG PET model **(A)**, 3DT1W MRI model **(B)**, and multimodal model **(C)**. Weight distribution map of the radiomics features in the [^18^F]FDG PET model **(D)**, 3DT1W MRI model **(E)** and multimodal model **(F)**.

**Figure 3:**
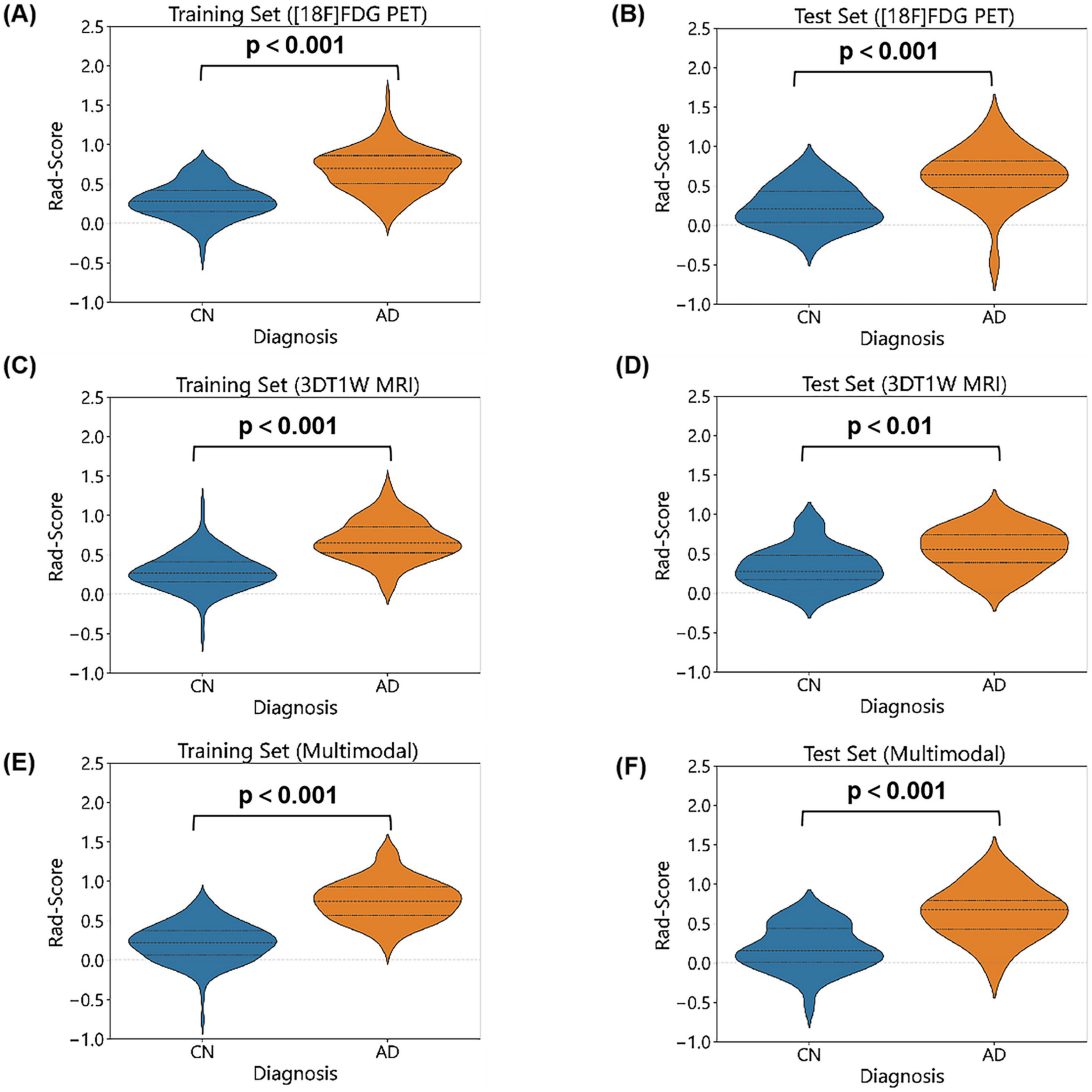
Comparison of Rad-Score between AD and CN groups. Rad-Scores of [^18^F]FDG PET model for AD and CN in the training set **(A)** and test set **(B)**. Rad-Scores of 3DT1W MRI model for AD and CN in the training set **(C)** and test set **(D)**. Rad-Scores of multimodal model for AD and CN in the training set **(E)** and test set **(F)**.

5,200 features were extracted from 26 cerebellar subregions for each modality. After feature selection using the Mann–Whitney U test, mRMR, and RFE, the [^18^F]FDG PET model, 3DT1W MRI model, and multimodal model retained 15, 15, and 20 features, respectively. Finally, LASSO regression identified 10, 13, and 15 non-zero coefficient features for the construction of the final machine learning models for each modality. The LASSO cross-validation error curve and coefficient profiles were presented in Supplementary Figure S1. The correlation heatmap and feature weight distribution were shown in Figure 2. The Rad-Score between AD and CN was statistically significant in all three modalities, as shown in Figure 3.

### 3.3 Model diagnostic performance

**Figure 4:**
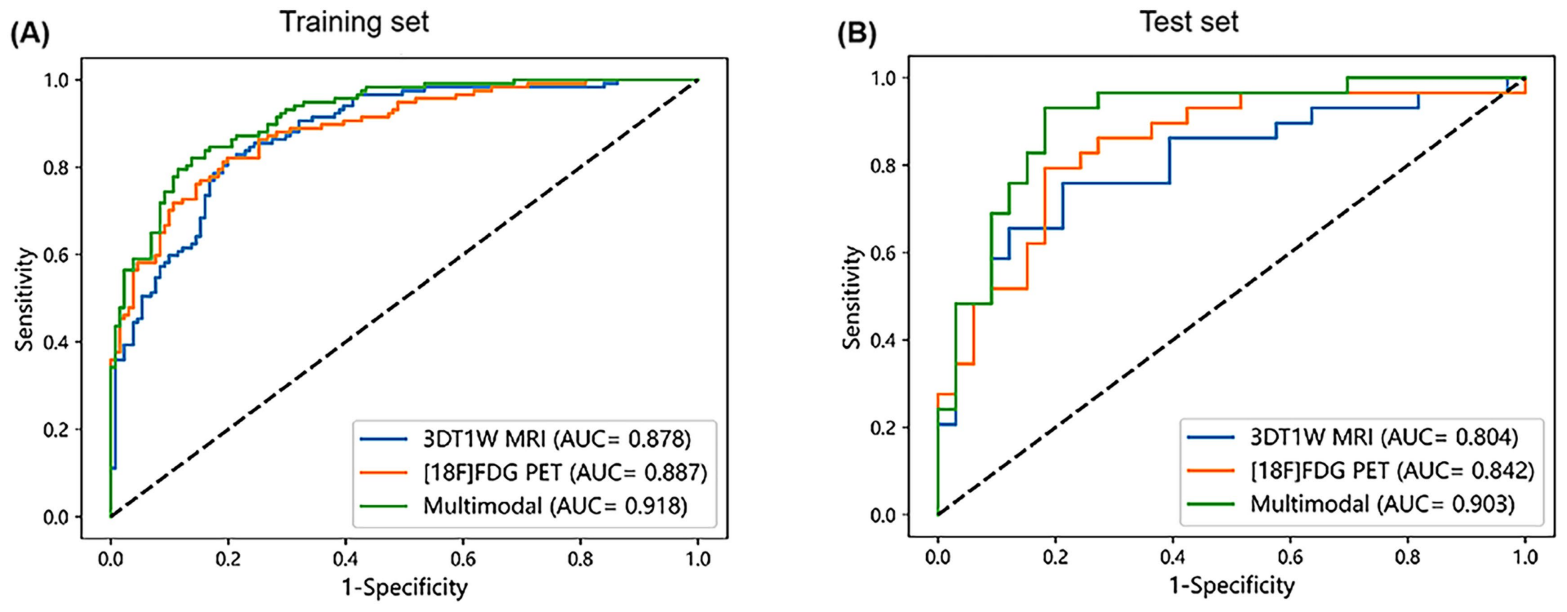
ROC curves for each model in the training **(A)** and test set **(B)**.

Performance evaluation was conducted for cerebellar single-modality ([^18^F]FDG PET, 3DT1W MRI) and multimodal ([^18^F]FDG PET combined with 3DT1W MRI) classification models. The results demonstrated that all three models effectively distinguish AD from CN subjects, and the multimodal model showed superior discriminative ability. As shown in Table 3 and Figure 4, the multimodal model achieved an AUC of 0.918 and an accuracy of 84.3% in the training set, and an AUC of 0.903 and an accuracy of 82.3% in the test set, both of which were higher than those of the [^18^F]FDG PET model (training set: AUC = 0.887, accuracy = 81.0%; test set: AUC = 0.842, accuracy = 79.0%) and the 3DT1WI MRI model (training set: AUC = 0.878, accuracy = 78.6%; test set: AUC = 0.804, accuracy = 74.2%). To statistically compare these results, we conducted DeLong’s test on the test set predictions. The AUC of the multimodal model was significantly higher than that of the [^18^F]FDG PET model (*z* = −2.536, *p* = 0.011). The improvement over the 3DT1W MRI model showed a strong trend toward significance (*z* = −1.735, *p* = 0.083). No significant difference was found between the two single-modality models (*z* = −0.557, *p* = 0.577).

**Table 3:** Performance of the single-modality and multimodal machine learning models.

	[^18^F]FDG PET		3DT1W MRI		Multimodal	
	Training set	Test set	Training set	Test set	Training set	Test set
AUC	0.887	0.842	0.878	0.804	0.918	0.903
95% CI	0.888–0.939	0.820–0.863	0.874–0.931	0.734–0.826	0.922–0.967	0.852–0.913
Accuracy	0.810	0.790	0.786	0.742	0.843	0.823
Sensitivity	0.769	0.828	0.735	0.586	0.821	0.793
Specificity	0.847	0.758	0.832	0.879	0.863	0.849
PPV	0.818	0.750	0.796	0.810	0.842	0.821
NPV	0.804	0.833	0.779	0.707	0.843	0.824
F1 score	0.793	0.787	0.764	0.680	0.831	0.807

3DT1W MRI, 3DT1-weighted magnetic resonance imaging; [^18^F]FDG PET, [^18^F]fluorodeoxyglucose positron emission tomography; AUC, the area under the curve; CI, confidence interval; NPV, negative predictive value; PPV, positive predictive value.

**Figure 5:**
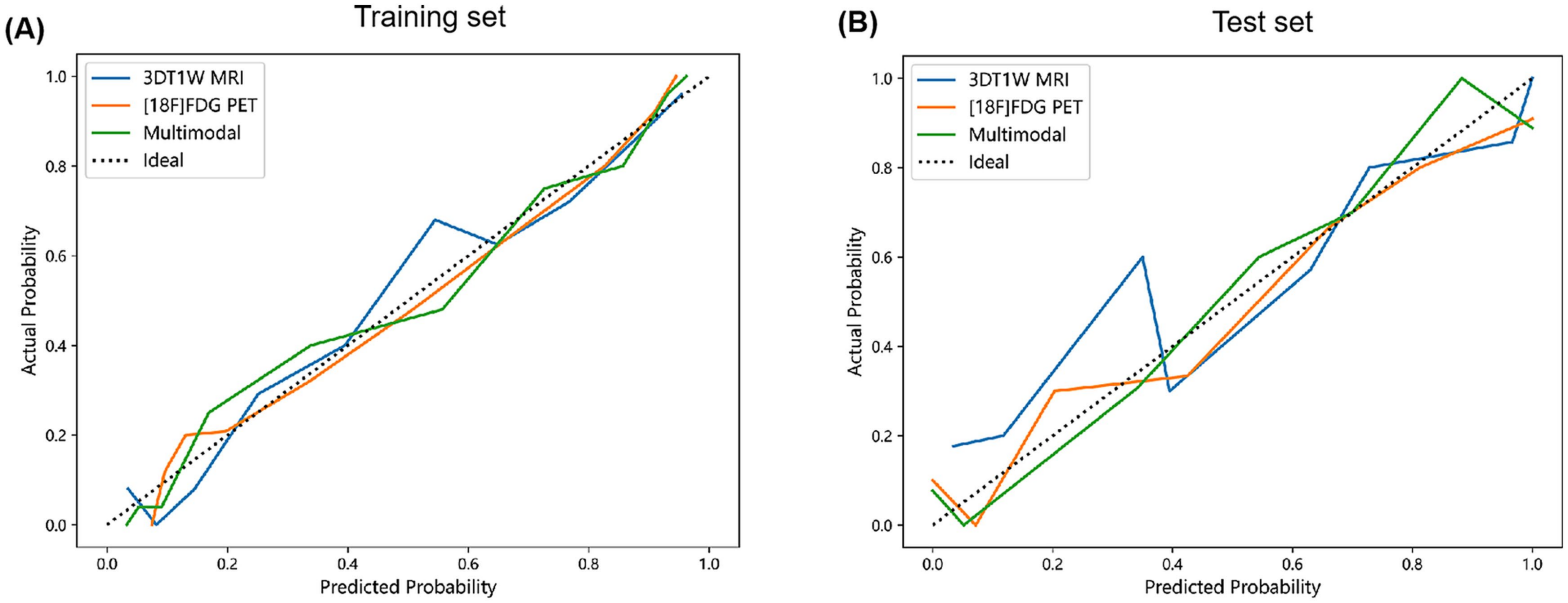
Calibration curves for each model in the training **(A)** and test set **(B)**.

**Figure 6:**
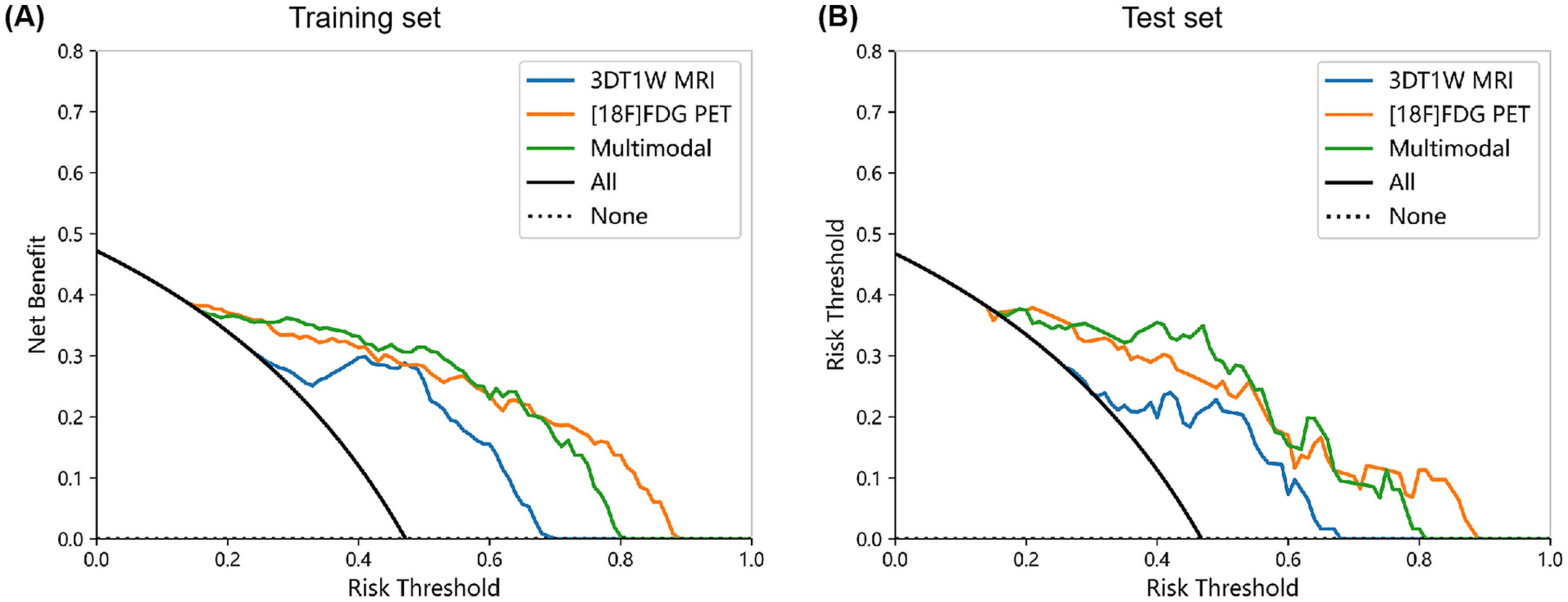
DCA for each model in the training **(A)** and test set **(B)**.

Model performance was further assessed by calibration curves and DCA, as shown in Figures 5, 6. The calibration curves showed that the predicted probabilities of the three models were in well agreement with the true observations in both the training and test sets (Hosmer-Lemeshow statistic, *p* > 0.05). The DCA indicated that all three models showed high clinical net benefit across the clinical application threshold range. Notably, the multimodal model seemed to exhibit stronger calibration ability and better clinical applicability compared to the single-modality models in the test set. These results suggested that the multimodal integration strategy can enhance the accuracy and practicality of early AD diagnosis.

### 3.4 Feature interpretation and visualization

**Figure 7:**
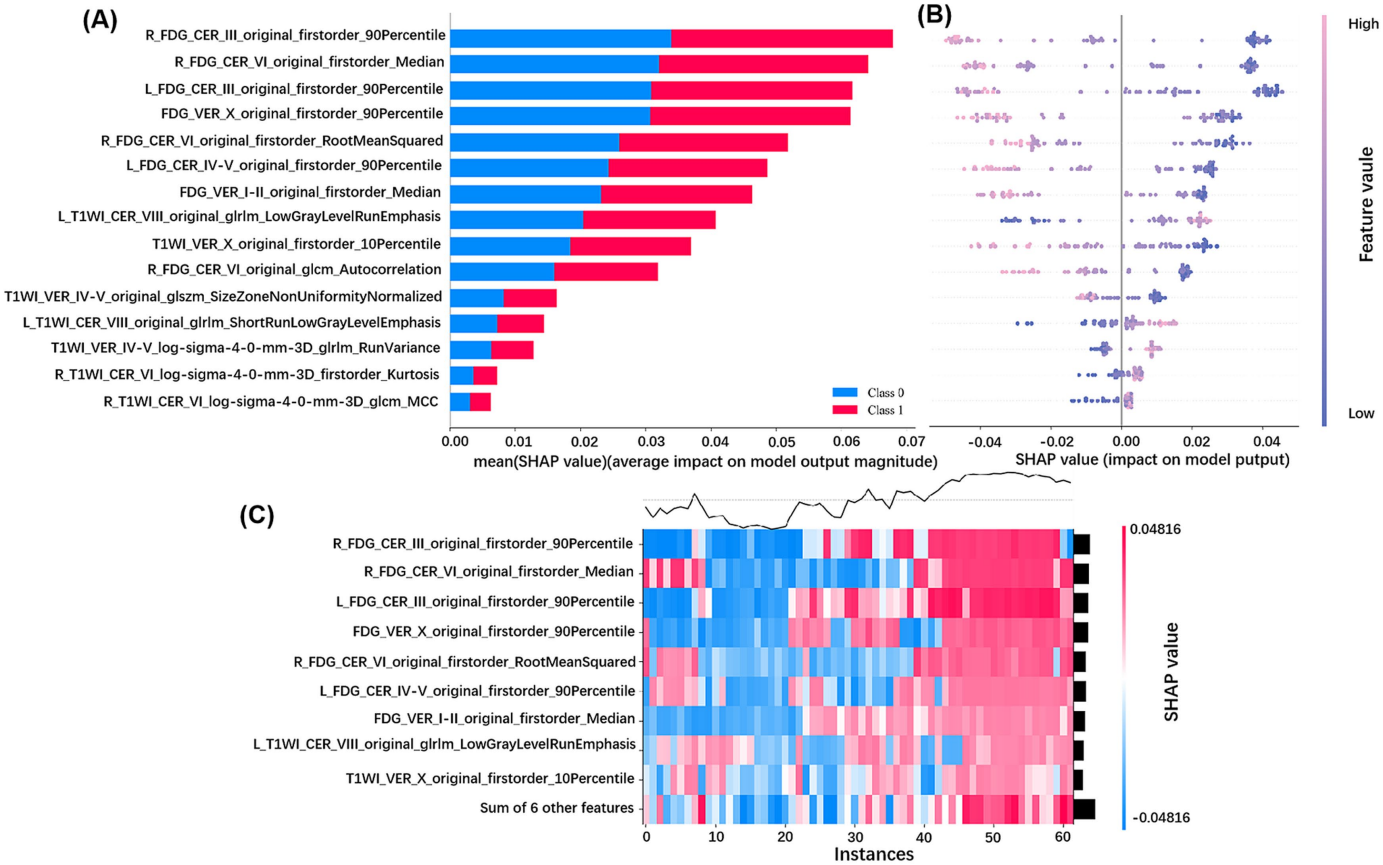
SHAP analysis of the multimodal model for distinguishing CN and AD. **(A)** Bar plot of mean SHAP values for radiomics features, ranking their average impact on the model’s AD - CN classification and showing direction of influence (Class 0: CN; Class 1: AD); **(B)** Bee - swarm plot of SHAP values for AD patients, illustrating how feature values affect the model’s output, with positive SHAP values driving AD classification and negative ones favoring CN; **(C)** Heatmap of SHAP values for key features across AD patients, visualizing the combined influence of features on individual instances, where red/blue intensity reflects the strength of promoting/inhibiting AD prediction.

We interpreted and visualized the radiomics features in the multimodal model by plotting SHAP bar plot, bee-swarm plot, and heatmap, as shown in Figure 7. Among the 15 features used for modeling, we found 7 features based on 3DT1W MRI and 8 features based on [^18^F]FDG PET, where the features from PET are more important for modeling. Specifically, R_FDG_CER_III_original_firstorder_90Percentile and R_FDG_CER_VI_original_firstorder_Median were the top two features, playing a key role in distinguishing AD from CN.

### 3.5 Robustness validation with multiple algorithms

**Figure 8:**
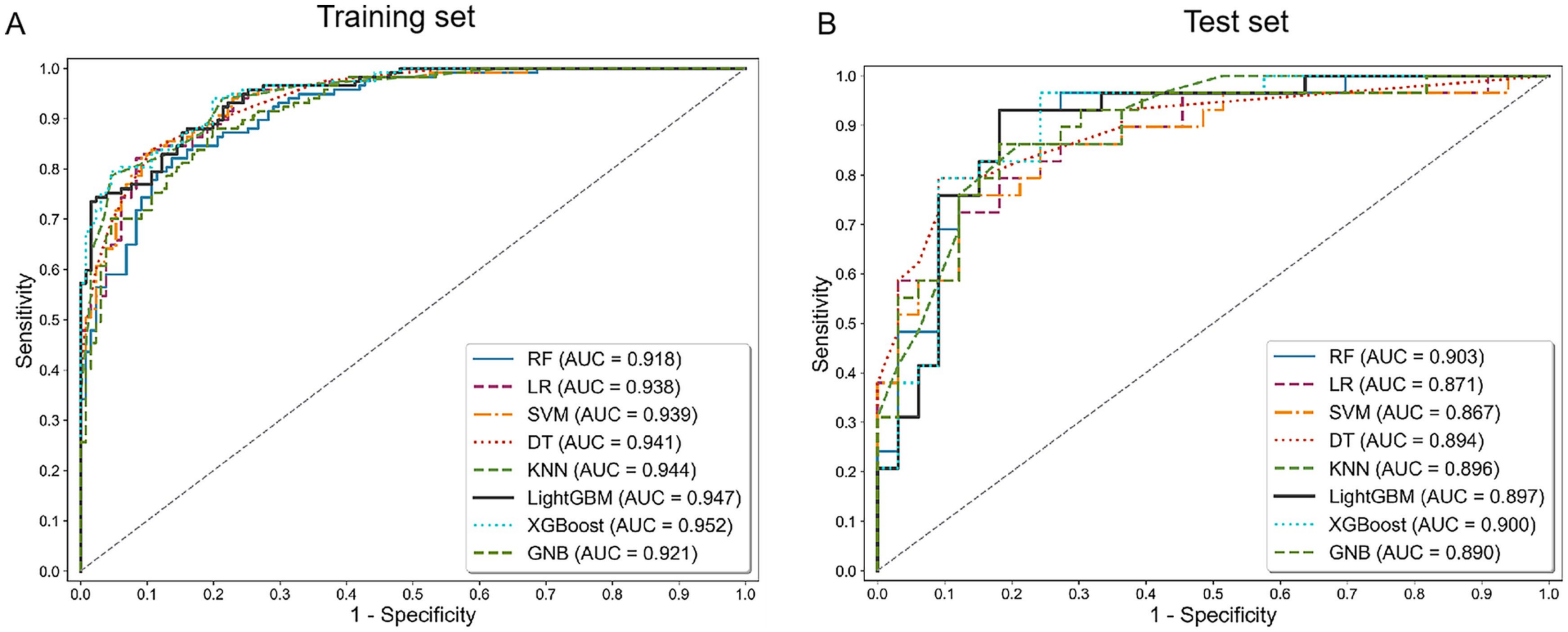
ROC curves for the multimodal model across all machine learning algorithms in the training **(A)** and test set **(B)**.

Overall, the diagnostic performance achieved using different machine learning algorithms was highly consistent with our primary results. All models constructed with these algorithms exhibited excellent performance, and multimodal models consistently outperformed single-modality models, with their AUC values ranging from 0.867 to 0.900 (Supplementary Table S1). Figure 8 and Supplementary Figure S2, respectively, present the ROC curves corresponding to all multimodal and single-modality models. The consistency in performance across different algorithms—from simple linear models to complex ensemble learning methods—provides strong evidence that the multimodal cerebellar radiomic features identified in our study inherently possess high discriminative power, thereby supporting robust and high diagnostic accuracy.

## 4 Discussion

This study is the first to comprehensively extract multimodal radiomic features from distinct cerebellar subregions, integrating complementary information from both metabolic and structural imaging to establish an objective machine learning classification model for distinguishing between CN and AD. Compared to the single-modality model, the multimodal model significantly improved the accuracy of AD recognition, achieving an AUC of 0.918 and an accuracy of 84.3% in the training set, and an AUC of 0.903 and an accuracy of 82.3% in the test set. SHAP analysis of the multimodal model showed that among the 15 selected features, the top seven features with the highest contribution all originated from the [^18^F]FDG PET images. Among them, R_FDG_CER_III_original_firstorder_90Percentile and R_FDG_CER_VI_original_firstorder_Median were the two most important features for distinguishing CN and AD, underscoring the unique value of cerebellar metabolic feature heterogeneity in AD.

Traditionally, the cerebellum has been considered primarily responsible for coordinating voluntary movements, maintaining postural balance, and regulating muscle tone. However, recent studies have demonstrated its extensive involvement in various cognitive processes, including executive function, language processing, working memory, and emotional regulation (Guell et al., 2018; Biondi et al., 2024). Direct damage to the cerebellum can lead to cerebellar cognitive affective syndrome, further emphasizing its critical role in higher cognitive functions and emotional modulation (Hoche et al., 2018). Anatomically, the cerebellum is divided into the anterior lobe (I-V), posterior lobe (VI-X), and the vermis. Among these, the anterior lobe primarily regulates motor functions, while the posterior lobe is involved in cognitive processing (Manto, 2022; Yang et al., 2024). This functional heterogeneity manifests as a specific pattern of impairment under disease conditions. Significant differences in cerebellar glucose metabolism patterns were found in patients with vascular cognitive impairment, with posterior lobe metabolism levels positively correlating with cognitive performance, while metabolism in the anterior lobe and vermis showed negatively correlated with cognition (Weng et al., 2025). In patients with AD, significant atrophy occurred in the vermis and paravermal regions of the anterior cerebellar lobe (I-V) and the posterior lobe (VI) during the MCI stage. However, as the disease progressed, the posterior lobe hemisphere (VII lobule) and Crus I were more prominently affected (Toniolo et al., 2018). Additionally, autopsy findings have confirmed significant atrophy of the granule cell layer in the lateral regions of the cerebellum in AD patients, with the degree of synaptic loss closely correlated with Braak stages (Samstag et al., 2025). These findings suggest that AD patients exhibit cerebellar-specific pathological changes and imaging abnormalities, which display regional heterogeneity. This provides a theoretical basis for the use of radiomics techniques to quantitatively analyze the structural and metabolic features of cerebellar subregions and subsequently develop a diagnostic model for AD. Our results demonstrated that the cerebellar subregion radiomics model, based on [^18^F]FDG PET combined with 3DT1W MRI, achieved excellent diagnostic performance for AD. The synergistic integration of multimodal data significantly improved model performance. Furthermore, the varying contributions of subregional features further highlighted the critical value of cerebellar subregional heterogeneity in the early diagnosis of AD.

SHAP analysis showed that the contribution of [^18^F]FDG PET metabolic image features was significantly higher than that of 3DT1W MRI structural image features in the multimodal model (all of the top seven features were derived from PET). The above indicates that cerebellar metabolic features extracted from PET images are more sensitive for AD diagnosis than structural features derived from MRI. Notably, most of the AD patients included in this study were at an early disease stage (MMSE score: 22.34 ± 4.05), further supporting the hypothesis that metabolic disturbances precede macrostructural changes (Jagust et al., 2006). This may be attributed to the ability of PET imaging to directly reflect the functional state of neuronal glucose metabolism, thereby capturing early pathophysiological activities such as synaptic dysfunction more effectively (Chételat et al., 2020). In contrast, structural MRI primarily captures gray matter atrophy, which often represents a later morphological consequence resulting from sustained neuronal injury (Pini et al., 2016). Consequently, during the early stages of the disease, PET-derived radiomic features can earlier and more directly detect AD-related neuronal abnormalities, which likely constitutes the fundamental reason for their superior discriminative power. These findings align with previous AD studies reporting that metabolic abnormalities in the temporoparietal cortex and hippocampus are early predictors for the conversion from CN to AD, exhibiting greater sensitivity than structural MRI (Ewers et al., 2014).

On the other hand, we identified two important radiomics features for AD diagnosis in the right cerebellar III and VI lobules on [^18^F]FDG PET images, namely R_FDG_CER_III_original_firstorder_90Percentile and R_FDG_CER_VI_original_firstorder_Median. The original first-order features are mainly used to capture the voxel intensity distribution within the region of interest, quantifying cerebellar metabolic levels from multiple dimensions and thereby indicating alterations in neuronal activity or synaptic function (Lambin et al., 2017). Lobule III, traditionally regarded as part of the anterior cerebellum that projects to the primary motor cortex, participates in motor regulation (Kelly and Strick, 2003). Metabolic abnormalities in this lobule may serve as a potential underlying cause of motor deficits (e.g., gait instability, bradykinesia) observed in AD patients, and patients with these motor symptoms have been shown to experience more rapid cognitive decline (Oveisgharan et al., 2024; Shaw et al., 2025). Notably, a recent large-scale multicenter study demonstrated that texture features in the right lobule III can predict the conversion from CN to MCI, and are significantly correlated with the severity of cognitive impairment across different levels of Aβ and p-tau pathology (Chen et al., 2025). Therefore, we speculate that metabolic abnormalities in lobule III not only reflect the early involvement of motor-related circuits in AD but also indicate that this region may serve as a motor–cognitive integration hub, playing a critical role in the multisystem dysfunction of AD. As a core cognitive subregion in the posterior cerebellar lobe, lobule VI participates extensively in higher cognitive functions via extensive connections with the default mode network and fronto-parietal control network (Henschke and Pakan, 2020; Buckner et al., 2011). fMRI studies have consistently demonstrated that bilateral lobule VI activation supports working memory, with the left hemisphere predominating in socioemotional processing and the right in language tasks, corresponding closely to the clinical manifestations of AD (Stoodley et al., 2012; Guell et al., 2018). Large-scale meta-analyses have further identified lobule VI as the most consistently reported cerebellar region exhibiting functional abnormalities and gray matter atrophy in both MCI and AD (Bernard et al., 2025; Gellersen et al., 2021; Colloby et al., 2014). The metabolic alterations of this lobule observed in our study provide new evidence for the essential role of Lobule VI in AD from the perspective of energy metabolism. This mechanism may result from direct damage to local neurons by AD pathology, or secondary metabolic decline due to weakened functional connectivity caused by degeneration of upstream associative cortices.

It is worth noting that in previous [^18^F]FDG PET studies, the cerebellum has commonly been used as a reference region for the normalization of cortical metabolism, leading to the long-term neglect of its intrinsic metabolic pattern in AD (Yan et al., 2020). Our findings raise an important question: does the cerebellum remain a scientifically appropriate reference region for metabolic normalization? Future studies should explore more deeply the specific metabolic patterns of subregions within the cerebellum in AD. On the other hand, the results of this study also reveal its potential for clinical application. The DCA curve showed that our model provides favorable clinical net benefit across a wide range of threshold probabilities, aiding the distinction between CN and AD and potentially optimizing diagnostic workflows. More importantly, the model is based on [^18^F]FDG PET and 3DT1W MRI—two modalities routinely acquired in clinical practice for AD—and requires no additional scanning sequences or equipment, which lowers the barrier for clinical translation. Although still at the research stage, this strategy points to a clear direction: in-depth secondary analysis of routine imaging can transform such imaging into high-value quantitative diagnostic biomarkers. The core of future work will be the development of standardized and automated analysis tools, with the ultimate goal of providing effective decision support for the early identification and intervention in AD.

Nevertheless, our study has some limitations. First, the relatively small sample size, with all data obtained from the ADNI database, may limit the generalizability of the model. Specifically, the small sample size may be insufficient to fully capture the inherent pathological heterogeneity of AD and might compromise the detection of low-abundance features in small cerebellar subregions. These factors may lead to imprecise estimation of model performance, and the stability of key features in a broader population requires further validation. While rigorous feature selection, independent test set partitioning, and cross-algorithm validation ensure internal robustness, external validation with independent multi-center cohorts remains essential. Second, the cross-sectional design limits the ability to analyze the dynamic evolution of cerebellar subregional features during AD progression or to evaluate their predictive value for conversion from MCI to AD, which requires follow-up studies using longitudinal cohorts. Finally, though the radiomics features showed strong diagnostic performance, their correlation with key AD molecular pathologies (such as Aβ deposition and tau tangles) remains unexplored and warrants further investigation.

## 5 Conclusion

In conclusion, this study is the first to integrate [^18^F]FDG PET metabolic images with 3DT1W MRI structural images to innovatively extract multimodal radiomics features from different cerebellar subregions, successfully constructing a highly accurate machine learning model for AD diagnosis. The model has demonstrated excellent performance and holds great potential for future clinical applications. Meanwhile, this study breaks the traditional paradigm of AD research centered on the cortex-hippocampus and holds promise for providing cerebellar-based potential biomarkers for clinical use.

## Data Availability

The original contributions presented in the study are included in the article/Supplementary material, further inquiries can be directed to the corresponding authors.

## Footnotes

^1^ http://adni.loni.usc.edu/

^2^ https://www.nitrc.org/projects/mricron/

^3^ https://www.python.org/

## Group member of the Alzheimer’s Disease Neuroimaging Initiative

Data used in preparation of this article were obtained from the Alzheimer's Disease Neuroimaging Initiative (ADNI) database (adni.loni.usc.edu/). As such, the investigators within the ADNI contributed to the design and implementation of ADNI and/or provided data but did not participate in analysis or writing of this report. A complete listing of ADNI investigators can be found at: http://adni.loni.usc.edu/wp-content/uploads/how_to_apply/ADNI_Acknowledgement_List.pdf.
