# Supplementary material for: Multimodal radiomics of cerebellar subregions for machine learning-driven Alzheimer’s disease diagnosis
Source: Front Aging Neurosci. 2025 Oct 27;17:1679788. doi: 10.3389/fnagi.2025.1679788 (PMC12598022; doi:10.3389/fnagi.2025.1679788)
Supplement: Supplementary file 1 [file Data_Sheet_1.PDF]

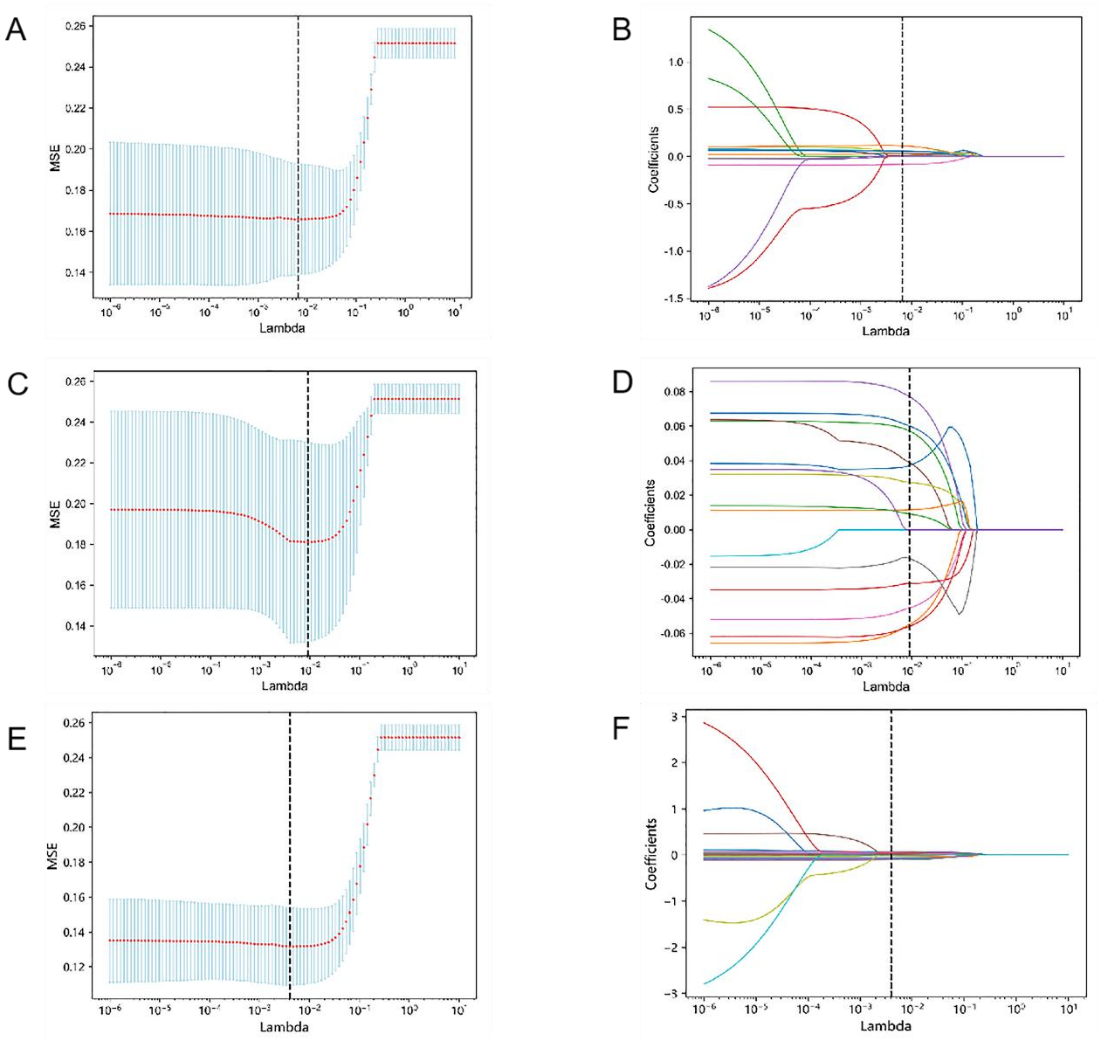

Figure S1. LASSO regularization for feature selection in different models. Cross-validation error curve (A) and coefficient profiles (B) of the [18F]FDG PET model; Cross-validation error curve (C) and coefficient profiles (D) of the 3DT1W MRI model; Cross-validation error curve (E) and coefficient profiles (F) of the multimodal model.

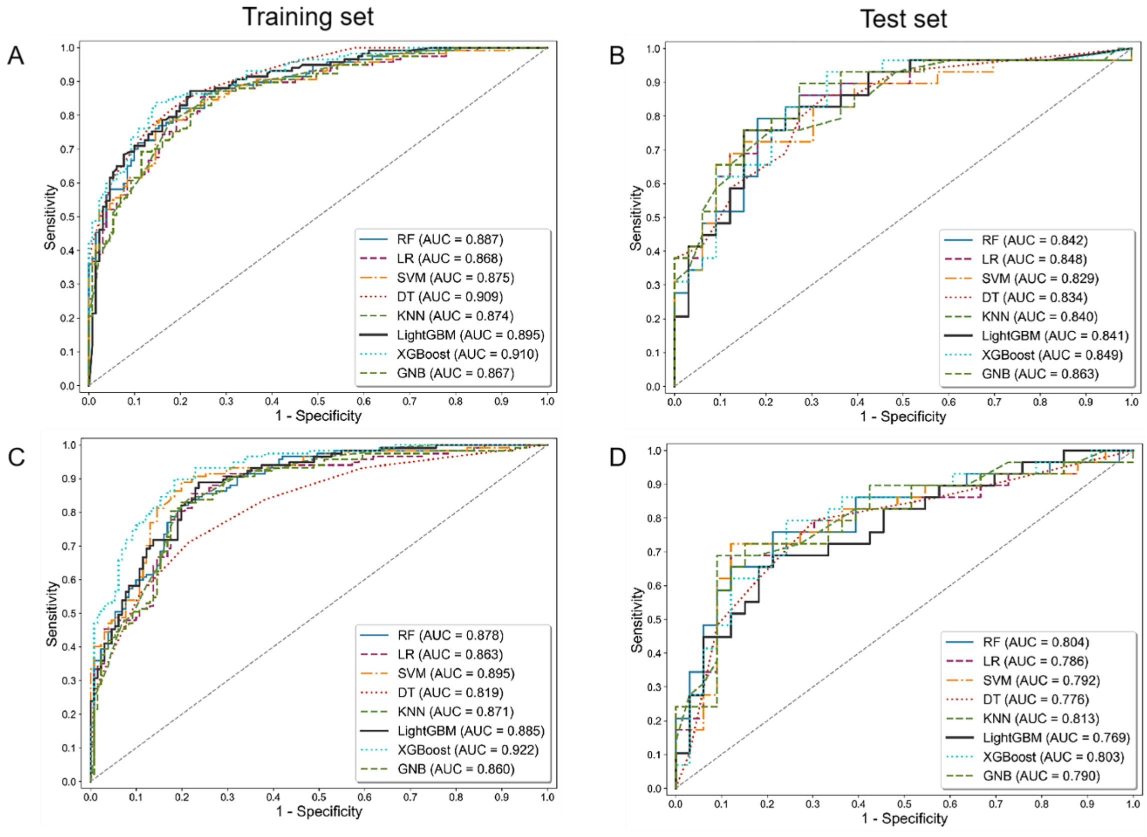

Figure S2. ROC curves across all machine learning algorithms. (A) [^{18}F]FDG PET model (training set); (B) [^{18}F]FDG PET model (test set); (C) 3DT1W MRI model (training set); (D) 3DT1W MRI model (test set).

Table S1 Performance of the single-modality and multimodal models across different machine learning algorithms

	Model	FDG PET		3DT1W MRI		Multimodal	
		Training set	Testing set	Training set	Testing set	Training set	Testing set
AUC	RF	0.887	0.842	0.878	0.804	0.918	0.903
	LR	0.868	0.848	0.863	0.786	0.938	0.871
	SVM	0.875	0.829	0.895	0.792	0.939	0.867
	DT	0.909	0.834	0.819	0.776	0.941	0.894
	KNN	0.874	0.840	0.871	0.813	0.944	0.896
	LightGBM	0.895	0.841	0.885	0.769	0.947	0.897
	XGBoost	0.910	0.849	0.922	0.803	0.952	0.900

	GNB	0.867	0.863	0.860	0.790	0.921	0.890
95% CI	RF	0.888-0.939	0.820-0.863	0.874-0.931	0.734-0.826	0.922- 0.967	0.852-0.913
	LR	0.853-0.885	0.845-0.851	0.843-0.916	0.727-0.824	0.917-0.971	0.853-0.875
	SVM	0.849-0.918	0.804-0.850	0.904-0.951	0.761-0.812	0.924-0.973	0.847-0.872
	DT	0.907-0.959	0.712-0.856	0.805-0.899	0.651-0.804	0.943-0.983	0.720-0.885
	KNN	0.845-0.913	0.784-0.855	0.824-0.917	0.764-0.835	0.916-0.968	0.853-0.904
	LightGBM	0.898-0.955	0.788-0.865	0.876-0.944	0.700-0.793	0.937-0.978	0.846-0.903
	XGBoost	0.913-0.961	0.800-0.863	0.923-0.972	0.727-0.813	0.952-0.983	0.864-0.909
	GNB	0.837-0.909	0.854- 0.866	0.829-0.918	0.759-0.813	0.887-0.951	0.878-0.904
Accuracy	RF	0.810	0.790	0.786	0.742	0.843	0.823
	LR	0.786	0.742	0.802	0.790	0.867	0.807
	SVM	0.798	0.790	0.831	0.807	0.863	0.823
	DT	0.819	0.726	0.750	0.726	0.867	0.790
	KNN	0.802	0.774	0.807	0.807	0.875	0.823
	LightGBM	0.811	0.807	0.786	0.710	0.851	0.823
	XGBoost	0.839	0.790	0.839	0.742	0.847	0.839
	GNB	0.778	0.790	0.811	0.758	0.827	0.807
Sensitivity	RF	0.769	0.828	0.735	0.586	0.821	0.793
	LR	0.744	0.759	0.778	0.690	0.838	0.724
	SVM	0.735	0.690	0.812	0.724	0.838	0.759
	DT	0.761	0.690	0.709	0.621	0.838	0.621
	KNN	0.778	0.759	0.803	0.690	0.786	0.759
	LightGBM	0.786	0.759	0.761	0.586	0.855	0.793
	XGBoost	0.812	0.828	0.829	0.621	0.846	0.828
	GNB	0.709	0.793	0.795	0.655	0.803	0.759
Specificity	RF	0.847	0.758	0.832	0.879	0.863	0.849
	LR	0.824	0.727	0.824	0.879	0.893	0.879
	SVM	0.855	0.879	0.847	0.879	0.886	0.879
	DT	0.870	0.758	0.786	0.818	0.893	0.939

	KNN	0.824	0.788	0.809	0.909	0.954	0.879
	LightGBM	0.832	0.849	0.809	0.818	0.847	0.849
	XGBoost	0.863	0.758	0.847	0.849	0.847	0.849
	GNB	0.840	0.788	0.824	0.849	0.847	0.849
PPV	RF	0.818	0.750	0.796	0.810	0.842	0.821
	LR	0.791	0.710	0.798	0.833	0.875	0.840
	SVM	0.819	0.833	0.826	0.840	0.867	0.846
	DT	0.840	0.714	0.748	0.750	0.875	0.900
	KNN	0.798	0.759	0.790	0.870	0.939	0.846
	LightGBM	0.807	0.815	0.781	0.739	0.833	0.821
	XGBoost	0.841	0.750	0.829	0.783	0.832	0.828
	GNB	0.798	0.767	0.802	0.792	0.825	0.815
NPV	RF	0.804	0.833	0.779	0.707	0.843	0.824
	LR	0.783	0.774	0.806	0.763	0.860	0.784
	SVM	0.783	0.763	0.835	0.784	0.859	0.806
	DT	0.803	0.735	0.752	0.711	0.860	0.738
	KNN	0.806	0.788	0.822	0.769	0.833	0.806
	LightGBM	0.813	0.800	0.791	0.692	0.867	0.824
	XGBoost	0.837	0.833	0.847	0.718	0.861	0.849
	GNB	0.764	0.813	0.818	0.737	0.828	0.800
F1	RF	0.793	0.787	0.764	0.680	0.831	0.807
	LR	0.767	0.733	0.788	0.755	0.856	0.778
	SVM	0.775	0.755	0.819	0.778	0.852	0.800
	DT	0.798	0.702	0.728	0.679	0.856	0.735
	KNN	0.788	0.759	0.797	0.769	0.856	0.800
	LightGBM	0.797	0.786	0.771	0.654	0.844	0.807
	XGBoost	0.826	0.787	0.829	0.692	0.839	0.828
	GNB	0.751	0.780	0.798	0.717	0.814	0.786
